# Short Sight, Long Sight, and Astigmatism : An Elementary Guide to the Refraction of the Eye

**Published:** 1887-09

**Authors:** 


*Short Sight, Long Sight, and Astigmatism: An Elementary Guide to the Refraction of the Eye.* By GEORGE F. HELM, M.A. Cantab., M.D. London: J. & A. CHURCHILL. 1886.

This book is intended to serve as an introduction to more advanced manuals on the same subject. It discusses the passage of rays of light through lenses. The method of using test-lenses in the different errors of refraction to which the eye is subject, is dealt with in an easy and practical manner, considerable space being allotted to astigmatism.

There are thirty-five diagrams in ninety-seven pages; the book is thus made easy for beginners. The subject matter is arranged in logical sequence, and what is stated is correct.

There is a great deal, however, that is left unsaid, and, taken by itself, this book can scarcely be recommended as providing the student or practitioner with a competent guide in refraction-cases.

